# Involvement of IL-13 and Tissue Transglutaminase in Liver Granuloma and Fibrosis after *Schistosoma japonicum* Infection

**DOI:** 10.1155/2014/753483

**Published:** 2014-07-03

**Authors:** Juanjuan Tang, Huayi Huang, Xiaofang Ji, Xunmin Zhu, Yinyan Li, Mingmin She, Suikai Yan, Mingchiu Fung, Zi Li

**Affiliations:** ^1^Guangzhou Hoffmann Institute of Immunology, School of Basic Sciences, Guangzhou Medical University, Guangzhou 510182, China; ^2^Morphology Department, School of Basic Sciences, Guangzhou Medical University, Guangzhou 510182, China; ^3^School of Life Sciences, The Chinese University of Hong Kong, Hong Kong

## Abstract

Schistosomiasis, one of the most devastating parasitic diseases, is caused by *Schistosoma japonicum* (*Sj*) infection resulting in serious liver fibrosis. Interleukin- (IL-) 13, which is produced by T_H_2  cells, is a critical profibrotic cytokine found in various organs, including the liver. Tissue transglutaminase (tTG), a group of multifunctional enzymes, serves a central function in the pathogenesis of chronic liver diseases. However, the relationship between IL-13, tTG, and liver fibrosis during *Schistosoma* infection has not been established. This study investigated the involvement of IL-13 and tTG in liver fibrogenesis during *Sj* infection in mice. Five weeks after *Sj* infection, granuloma and fibrosis development in the liver coincided with an increase in IL-13 and tTG in the liver and the upregulation of serum IL-13 in infected mice. Administration of cystamine, an inhibitor of tTG, abrogated the increase in both tTG and IL-13 in infected mice and ameliorated liver fibrogenesis and granuloma development. This result establishes a novel link among IL-13, tTG, and liver granuloma and fibrosis under *Sj* infection. Based on their important functions in liver fibrosis induced by *Sj* infection, IL-13 and tTG could be promising potential drug targets against schistosomiasis.

## 1. Introduction

Despite decades of effort,* Schistosoma japonicum* (*Sj*) remains to be a prevalent* Schistosome* in Asia and is the only* Schistosome* causing infection in China. Although fundamental control has been achieved through potent governmental measures,* Sj* infection remains widespread and cannot be effectively controlled in some lakes and marshland regions, thus inflicting massive social and economic burdens [1–3]. Progressive liver fibrosis is one of the major hallmarks of* Sj* infections and accounts for the further development of portal hypertension, ascites, hepatosplenomegaly, or even liver cirrhosis [4]. However, the mechanism behind schistosomal liver fibrogenesis and immune response remains poorly understood.

As* Schistosome* infection progresses into the chronic phase,* Schistosome* eggs that are deposited in tissues begin to secrete soluble egg antigen (SEA), which suppresses Th1-mediated response and promotes Th2-mediated inflammatory reactions [4]. Thus, Th2-related cytokines are inferred to participate actively in fibrogenesis and have thus been extensively studied [5, 6]. Interleukin- (IL-) 13 is a cytokine secreted by several cell types, including eosinophils, mast cells, basophils, epithelial cells, smooth muscle cells, fibroblasts, macrophages, and T cells, especially Th2 cells. IL-13 participates in asthma, tumorigenesis, and parasitic diseases. In* Schistosoma mansoni-* (*Sm-*) infected mice, IL-13 mediated liver fibrosis as a promoter and sustainer [7]. Liver fibrogenesis is severely decreased in* Sm*-infected IL-13-deficient mice, as well as in wild-type animals treated with IL-13 antagonists, whereas in TGF-*β*1 and MMP-9 deficient mice, such alleviation effect is not apparent [8–10].* Sj* has pathological patterns similar to those of* Sm*, but no data have shown that IL-13 serves a function in the upstream regulation mechanism of liver fibrogenesis caused by* Sj* infection.

Tissue transglutaminase (tTG), as a multifunctional protein, performs a variety of intracellular and extracellular functions, including hydrolyzing GTP, transamidation activity as a catalyzing enzyme, and cross-linking glutamine residues and lysine residues of proteins. These properties cause tTG to be involved in various physiological processes, which, if regulated inappropriately, can also lead to its involvement in a number of diseases, such as metastatic cancer, coeliac disease, and lung, renal, and liver fibrosis [11–13]. tTG is overexpressed in both the murine experimental liver fibrosis model and hepatitis c virus-induced human liver fibrosis. RNA interference can result in diminution of liver fibrosis and lesser aggregation of fibrotic tissue [14]. Cystamine (CTM) can modify cysteine at the active site on tTG in a disulfide interchanging manner, such that the activity of tTG is selectively inhibited [15]. Inhibition of tTG activity by CTM results in the diminution of liver fibrosis induced by CCl_4_ [16]. Meanwhile, tTG knockout mice with CCl_4_ intoxication display high lethality as compared with wild-type controls [17]. Therefore, enhanced tTG level seems to protect the liver from acute and chronic injury, but its net effect on fibrogenesis resulting from different causes requires further study.

Several studies have indicated that tTG serves as an important inflammatory and fibrogenetic factor [18]. tTG modulates inflammation, exerting both pro- and anti-inflammatory effects, such as positive feedback loop with TGF-*β*1 [19]. NF-*κ*B, a key inflammation-associated factor, is activated by tTG through the cross-linking of the C-terminal glutamine cluster of I*κ*Ba [20]. Epithelial tTG can induce Th17 differentiation and subsequent IL-17 production and pulmonary fibrosis in bleomycin-treated mice. Moreover, tTG reportedly regulates Th2 cytokine secretion and mediates in vitro and in vivo allergic inflammation [20, 21]. tTG can also downregulate peroxisome proliferator-activated receptor *γ* (PPAR*γ*), as well as increasing the classic parameters of inflammation, such as TNF-alpha, tyrosine phosphorylation, and MAPKs, in a model of cystic fibrosis [22].

In this study, we take an important step toward answering these questions by uncovering a novel connection among tTG, IL-13, and* Sj*-caused liver fibrosis. We find that tTG and IL-13 levels are upregulated in mice with* Sj* infection. Importantly, we further demonstrate that the treatment of mice with* Sj*-induced liver fibrosis by using an inhibitor of the enzymatic activity of tTG inhibits the* Sj*-induced upregulation of IL-13 and alleviates liver fibrosis. To our knowledge, these findings show that tTG is involved in the development of* Sj* infection-induced liver fibrosis in mice, and the mechanism may be associated with tTG-regulated IL-13 expression.

## 2. Methods

### 2.1. Animal Grouping, Parasite Infection, and Cystamine Administration

This study was performed in strict accordance with the recommendations in the Guide for the Care and Use of Laboratory Animals of State Scientific and Technological Commission. The protocol was approved by the Committee on the Ethics of Animal Experiments of Guangzhou Medical University. All surgeries were performed under sodium pentobarbital anesthesia, and every effort was made to minimize suffering.

Six- to eight-week-old female BALB/C mice (Experimental Animal Center of Sun Yat-Sen University, Guangzhou, China) were infected percutaneously through the abdomen with 20 ± 3* Sj* cercariae. The mice livers were collected and determined at weeks 5, 6, 8, and 12 after infection. Concurrent controls were uninfected mice.

Cystamine (CTM, Sigma-Aldrich, St. Louis, USA) is a common tTG inhibitor [15, 16, 23, 24]. In this study, for the mice group with tTG inhibitor-CTM treatment, starting at day 3 after infection, mice were administered with 100 *μ*L of CTM (10^−2^ mM, Sigma) in phosphate-buffered solution (PBS) through intraperitoneal injection once per day for 7 d, whereas the infection control group received PBS alone. Two noninfected control mice groups were treated with CTM or PBS. The number of mice in every group is 8. Sera of mice of all groups were collected for underlying analysis.

### 2.2. Calculation of Collagen Area Using Masson's Trichrome (MT) Staining

Fresh liver tissues were fixed in 4% paraformaldehyde overnight and routinely paraffin-embedded. Paraffin sections (4 *μ*m) were prepared from each liver tissue sample. The liver tissue sections were stained by MT staining to evaluate collagen content and distribution. The collagen fibers were stained blue, cell nuclei were stained black, and the background was stained red. Percentage of total areas with collagen positive color (blue) [percentage of collagen area (positive blue color area/total area)%] was analyzed using Image-Pro Plus 6.0 software. Every stained sample was evaluated in double-blind fashion by two independent investigators.

### 2.3. RNA Isolation and RT-PCR and Quantitative-PCR

Total RNA was extracted from fresh liver tissue homogenized in TRIZol reagent (Invitrogen) according to the manufacturer's protocol. RNA purity and concentration were assessed by spectrophotometry. Reverse transcriptase (RT) reactions for cDNA synthesis were performed using PrimeScript RT Master Mix (TAKARA). Relative mRNA expression level was determined by real-time quantitative polymerase chain reaction (Q-PCR) with SYBR Green I PCR Master (TAKARA) kit on ABI 7500 machine according to the manufacturer's protocol. The primers used were as follows: mouse-IL-13 (forward: CGGCAGCATGGTATGGAGTG, reverse: ATTGCAATTGGAGATGTTGGTCAG); mouse-tTG (forward: 5′-GTGAGCCGTGCTATCTGTCCTG-3′, reverse: 5′-ACTGCCTGCTTGGAACCTGAA-3′); mouse-GAPDH (forward: 5′-TGTGTCCGTCGTGGATCTGA-3′, reverse: 5′-TTGCTGTTGAAGTCGCAGGAG-3′). The Q-PCR results were expressed as fold amplification using the 2^−ΔΔCt^ method, with mouse GAPDH as the internal control. Each experiment was repeated thrice.

### 2.4. Measurement of Mice Sera IL-13 and Hyaluronic Acid by ELISA

Peripheral venous blood was collected by cutting the tail veins of mice. After being incubated in room temperature for 1 h, blood samples were centrifuged (1000 g, 4°C) for 10–15 min. Supernatant was extracted to detect IL-13 (Sigma-Aldrich, St. Louis, USA) and hyaluronic acid (Mouse Hyaluronic acid ELISA kit, Shanghai Yueyang Biological Technology Co., Ltd.) through ELISA measurement. Absorbance of the samples was measured at a 450 nm wavelength.

### 2.5. Immunohistochemistry (IHC) Assay

After sacrifice, the mouse livers were immediately fixed in 4% paraformaldehyde and then paraffin-embedded. Liver sections of 4 *μ*m were prepared. Endogenous peroxidase was blocked with 3% hydrogen peroxide (H_2_O_2_). Anti-IL-13 (AF-413-NA, R&D Systems), antismooth muscle actin alpha (*α*-SMA) (BM0002, BOSTER), or anti-tTG (sc-20621, Santa Cruz Biotechnology) primary antibody was diluted in 50-fold. IL-13, *α*-SMA, and tTG expressions were detected using GTVision II Detection System/Mo&Rb (Gene Tech (Shanghai) Co., Ltd.) according to the manufacturer's instructions.

### 2.6. Western Blot Analysis

Fresh mouse livers were ground into powder in liquid nitrogen, and moderate protein lysis solution (RIPA : PMSF = 100 : 1) was added. After incubation on ice for 30 min, tissue debris was removed by centrifugation (15 min, 4°C). Protein concentrations were assayed by using the Bradford assay (BIO-RAD). Total protein was resolved by SDS-PAGE and then transferred to a polyvinylidene fluoride membrane (0.2 *μ*m, Millipore). After blocking with 5% skimmed milk, membranes were probed with the appropriate antibody. Protein bands were detected with ECL reagents. The antibodies used were as follows: anti-GAPDH Ab (Cell Signaling Technology), anti-*α*-SMA (BM0002, BOSTER), anti-*α*-SMA (BM0002, BOSTER), and anti-tTG Ab (sc-20621, Santa Cruz Biotechnology).

### 2.7. Total Transglutaminase Activity in Lysates of Liver Tissue

The transglutaminase (TGase) activity of liver tissue lysates was determined through a modified nonradioactive microtiter plate assay. Briefly, the microtiter plates were coated with 100 *μ*L of* N,N*′-dimethylcasein (Sigma, 10 mg/mL to 20 mg/mL) at 4°C overnight, and the wells were blocked with nonfat dry milk (0.5% in 0.1 M Tris-HCl, pH 8.5) for 30 min. The wells were then washed twice with 350 *μ*L of 0.1 M Tris-HCl (pH 8.5). The following reagents were added to each well to obtain a total volume of 50 *μ*L per well: 5 mM CaCl_2_, 10 mM dithiothreitol, 0.5 mM 5-(biotinamido)pentylamine, 0.4 *μ*g lysates of liver tissue, and 0.1 M Tris-HCl (pH 8.5). The microtiter plate was incubated at 37°C for 30 min. The liquid was then discarded, and the reaction was stopped by washing twice with 350 *μ*L of 5 mM of EDTA, followed by washing twice with 350 *μ*L of 0.1 M Tris-HCl (pH 8.5). Streptavidin-horseradish peroxidase conjugates were diluted at a proportion of 1 : 200 with nonfat dry milk (0.5% in 0.1 M Tris-HCl (pH 8.5)) prior to the addition of 60 *μ*L of solution per well to be incubated for l h at room temperature. The plate was washed once with 350 *μ*L of 0.001% Triton X-100 followed by washing for four times with 350 *μ*L of 0.1 M Tris-HCl (pH 8.5). Then, 100 *μ*L of substrate solution (TMB, Sigma T0440) was added to each well. After incubation at room temperature for 10 min, the reactions were stopped by the addition of 25 *μ*L of 3 N HCl to each well, and the proteins into which the 5-(biotinamido)pentylamine was incorporated were quantified by measuring the absorbance at 450 nm in a plate reader (BioTek). The relative TGase activity was determined using the absorbance at 450 nm (OD450).

### 2.8. Statistics

All statistical analyses were performed using SPSS13.0 software. Statistical significance (*P* < 0.05) between the means of two groups was determined using Student's *t*-tests. Statistical comparisons of the means of multiple (>2) groups were determined using repeated-measure one-way ANOVA. All data were expressed as means ± SD, and all experiments were repeated twice or thrice.

## 3. Results

### 3.1. IL-13 Correlated with Hepatic Fibrosis after* Sj* Infection

**Figure 1:**
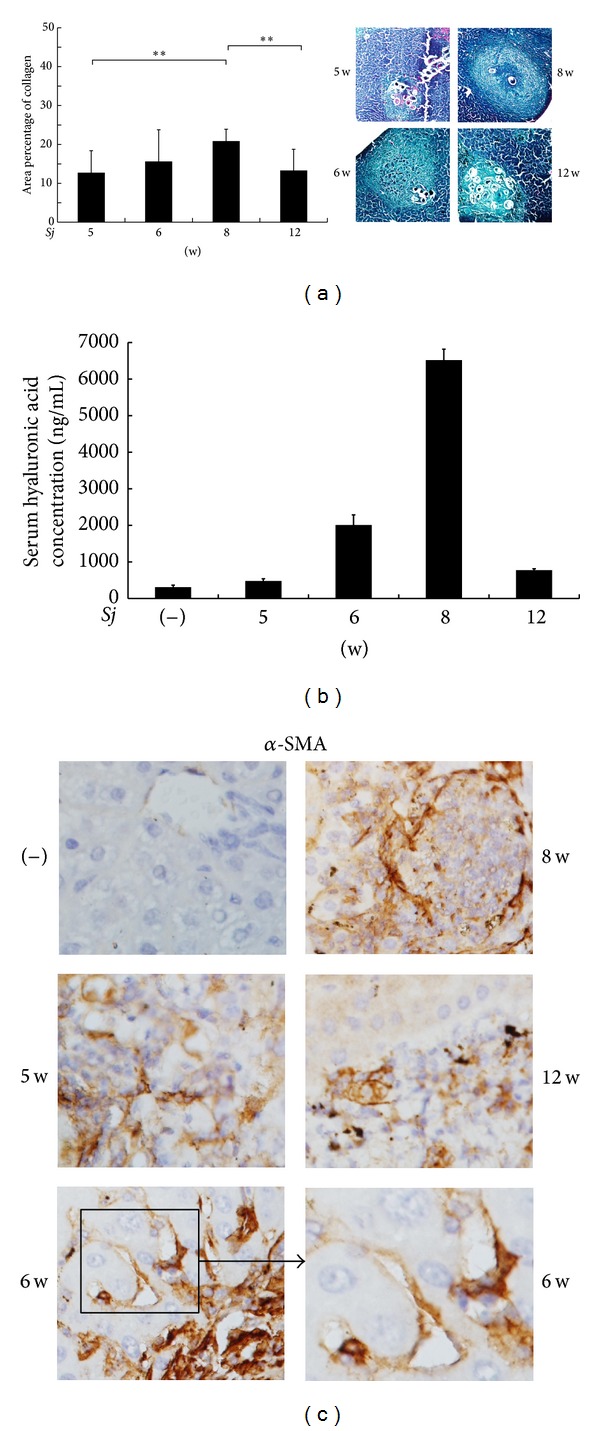
Extent of mouse hepatic fibrosis increased gradually after* Sj* infection. BALB/c mice were infected with 20 ± 3 infective cercariae of* Sj* for 5, 6, 8, and 12 weeks, and noninfected mice served as negative control. Liver tissues were fixed and stained with Masson trichrome or anti-*α*-SMA antibody (original magnification 40x). Collagen deposition area percentage (Masson trichrome staining positive area percentage of each section) was calculated and shown in (a, left panel). Representative liver granulomas stained with Masson's trichrome staining are shown in (a, right panel) at 20x magnification. Concentration of hyaluronic acid in mouse liver serum is shown in (b) through ELISA assay. Expression level of *α*-SMA protein in mouse liver tissues is shown in (c) through IHC assay. Data are presented as mean ± SD from eight mice per group. Experiments were performed twice. **P < 0.01.

**Figure 2:**
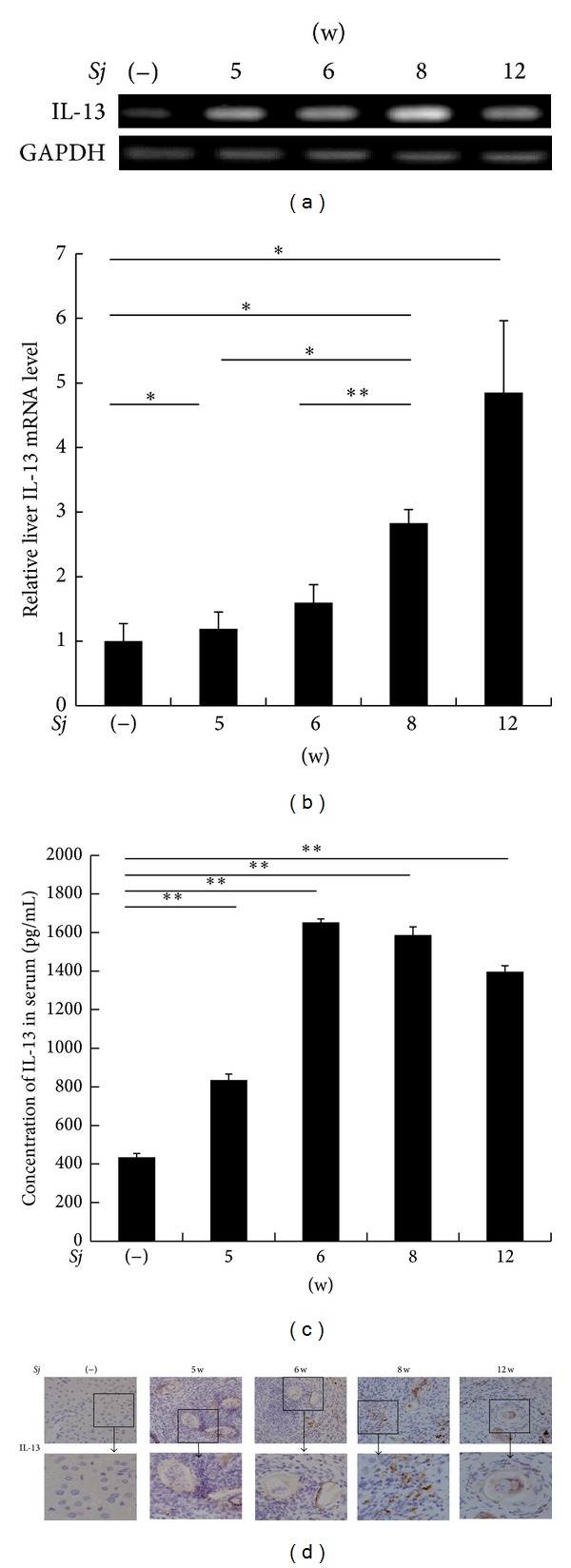
IL-13 level was associated with hepatic fibrosis after* Sj* infection. (a, b) IL-13 mRNA level in BALB/c mice liver is upregulated at weeks 5, 6, 8, and 12 after* Sj* infection, as determined through RT-PCR and SYBR Green based Q-PCR. GAPDH is detected as an internal control. Data are presented as mean ± SD from eight mice per group. *P < 0.05 and **P < 0.01. (c) Concentration of IL-13 in mice sera is tested by ELISA. Data are presented as mean ± SD from eight mice per group. *P < 0.05 and **P < 0.01. (d) Localization and expression level of IL-13 protein in BALB/c mice liver is shown through IHC assay at weeks 5, 6, 8, and 12 after* Sj* infection. Representative IL-13 staining sections are shown at primary 40x magnification.

BABL/c mice were infected cutaneously with 20 ± 3* Sj* cercariae of Chinese mainland strain, and mice liver tissues were collected at weeks 0, 5, 6, 8, and 12 after infection with* Sj*. Liver granuloma and fibrosis were evaluated by monitoring the liver manifestation, area of collagen fiber (stained blue in MT staining) (Figure 1(a)), level of serum hyaluronic acid (Figure 1(b)), and strength of *α*-SMA-positive staining as a marker of hepatic stellate cell (HSC) activation (Figure 1(c)).* Sj* liver granuloma began at week 5, and then fibrosis progressed the most seriously at week 8, whereas chronic liver fibrosis appeared at week 12. To evaluate the correlation between IL-13 and liver fibrogenesis, hepatic IL-13 expression profile in both* Sj*-infected mice and normal control was detected by RT-PCR. Gel analysis result showed that IL-13 maintained a relatively low basal expression in the liver of normal control mice but increased significantly after* Sj* infection (Figure 2(a)). This phenomenon was verified further by Q-PCR using the same RNA specimens (Figure 2(b)) and by ELISA to test serum IL-13 levels (Figure 2(c)). These results showed that, in response to* Sj* infection, IL-13 was highly expressed whether in liver tissue or peripheral venous blood. IL-13 did not reach a high level of expression until the infection advanced into its late phase, when egg-laying commenced [25]. This finding was in accordance with that of previous studies. The mRNA level of IL-13 began to drastically increase since week 5 and reached as high as fivefold in week 12 compared with the noninfected control. We also performed an IHC assay to monitor IL-13 expression in liver tissue (Figure 2(d)). At week 5, the extracellular matrix was distorted, and cells in liver tissue became disorganized, indicating the beginning of granuloma formation and hepatic fibrosis (Figure 1). During this time, IL-13 began to be detected in these damaged tissues (Figure 2(d)). This observation signified the possible function of IL-13 in granuloma formation and in the early stage of hepatic fibrogenesis. An interesting finding was that a high level of IL-13 appeared around the granuloma in the area of inflammatory cell infiltration, especially at week 8 after* Sj* infection, but cells around the veins where* Sj* adult worms were located did not exhibit IL-13 positive staining (Figure 2(d)). Given the central function of granuloma in hepatic fibrosis, these findings implied that IL-13 around the* Sj* egg granuloma was induced by the host inflammatory response to the stimulation of* Sj* SEA and, thus, accelerated granuloma formation and hepatic fibrosis in* Sj* infection.

### 3.2. tTG Contributes to a High Extent of Hepatic Fibrosis after* Sj* Infection

**Figure 3:**
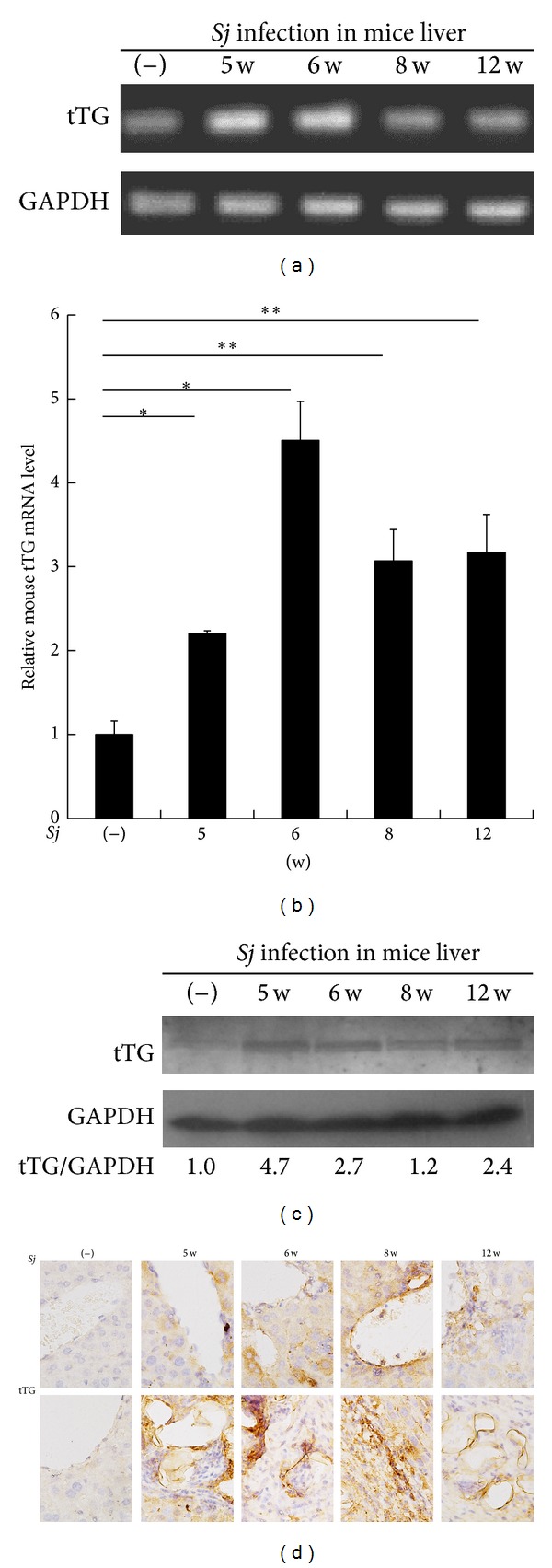
tTG expression was upregulated progressively in mice liver after* Sj* infection. (a, b) tTG mRNA level in BABL/c mice liver is upregulated at weeks 5, 6, 8, and 12 after* Sj* infection, as determined through RT-PCR and Q-PCR. GAPDH is detected as an internal control. Data are presented as mean ± SD from eight mice per group. *P < 0.05 and **P < 0.01 compared with noninfected mice (−). (c) tTG protein level of mice liver homogenates is tested by Western blot analysis. GAPDH is used as loading control. (d) Different localization expression levels of tTG protein in BALB/c mice liver are shown through IHC assay at weeks 5, 6, 8, and 12 after* Sj* infection. Upper panel: extent of tTG positive staining in hepatic cell and liver tissue around the liver sinusoid. Lower panel: extent of tTG positive staining in the granuloma and nearby liver tissue after* Sj* egg deposition. Representative tTG staining sections are shown at 40x magnification.

The involvement of tTG in liver fibrosis has long been suggested [13, 14, 16, 17], and our findings are consistent with those of numerous past experiments. We sacrificed mice at weeks 0, 5, 6, 8, and 12 after* Sj* infection. The RT-PCR and Q-PCR results showed that tTG mRNA expression levels were markedly increased in the liver after* Sj* infection compared with uninfected mice (Figures 3(a) and 3(b)), as well as tTG protein expression level (Figure 3(c)) and total TGase activity (see Figure S1 in Supplementary Material available online at http://dx.doi.org/10.1155/2014/753483). Furthermore, in* Sj*-infected mouse liver sections, tTG expression was found in the hepatic cells around the hepatic sinusoids where* Sj* adult worms were located or around and in the liver granuloma and fibrosis areas where* Sj* eggs were deposited (Figure 3(d)).

**Figure 4:**
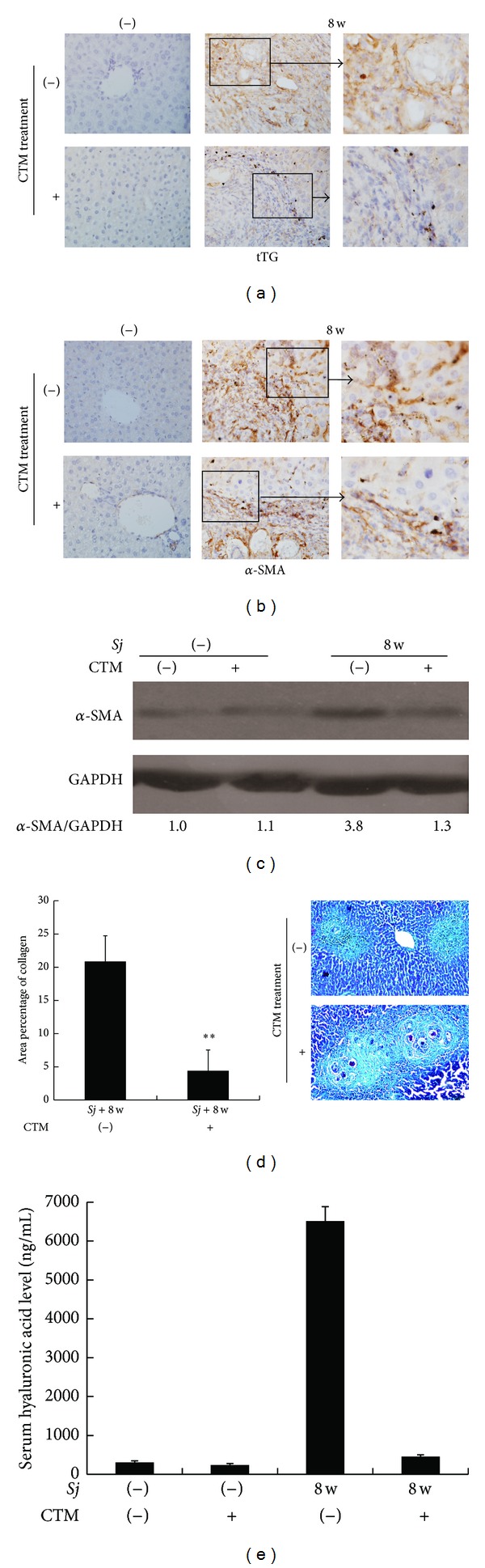
Cystamine as an inhibitor of tTG alleviated the extent of liver fibrosis. tTG activity in BALB/c mice is blocked by CTM intraperitoneal injection from day 3 to day 10 after* Sj* infection. Mice are sacrificed at week 8 after infection. Liver tissues are fixed and stained with anti-tTG (a), anti-*α*-SMA antibody (b) (original magnification 40x), or Masson trichrome (d) (magnification 20x). Noninfected mice with or without CTM treatment acted as controls. Collagen deposition area percentage (Masson trichrome staining positive area percentage of each section) is calculated and shown in (d). Level of *α*-SMA protein expression in mouse liver tissues is shown through Western blot analysis, and GAPDH is detected as an internal control (c). Concentration of hyaluronic acid in mouse liver serum is shown in (e) through ELISA assay. Data are presented as mean ± SD from eight mice per group. **P < 0.01.

To provide further evidence that tTG affects the development of* Sj*-infected mouse liver fibrosis, we treated the infected mice by using an intraperitoneal injection of CTM once per day for 7 d starting at day 3 after infection. Considering that liver fibrosis was the most severe at week 8 after infection (Figure 1), we sacrificed the CTM-treated infected mice and the infected mice without CTM treatment at week 8. Liver fibrosis was evaluated by monitoring the area of collagen deposition, serum hyaluronic acid level, and strength of *α*-SMA-positive staining by IHC assay. Compared with those of untreated mice, the liver tissues of CTM-treated* Sj*-infected mice showed significantly lower tTG expression (Figure 4(a)) and TGase activity (Figure S2), as well as *α*-SMA expression level (Figures 4(b) and 4(c)), the area size of collagen deposition (*P* < 0.001) (Figure 4(d)), and serum hyaluronicacid level (Figure 4(e)). The results indicated that tTG contributes to hepatic fibrosis after* Sj* infection.

### 3.3. Cystamine Partially Downregulated the Level of IL-13 in Response to* Sj* Infection

**Figure 5:**
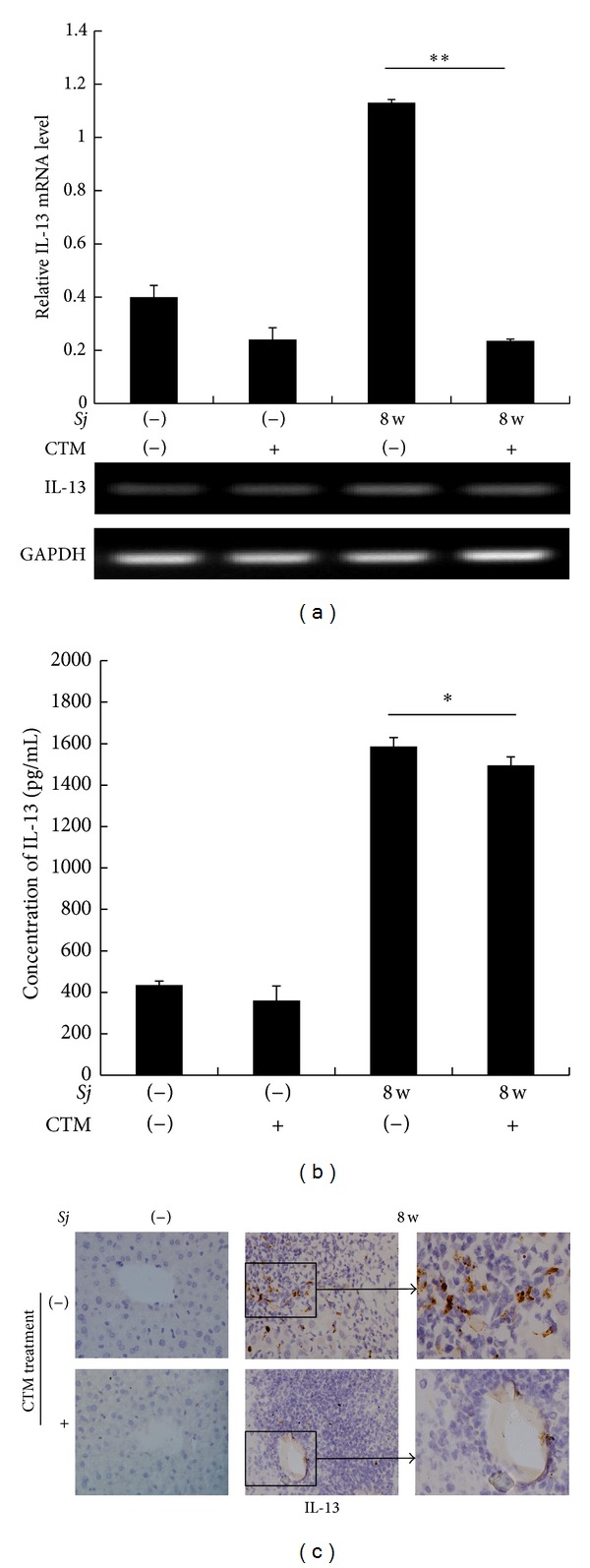
CTM reduced IL-13 expression profile in the mice liver during* Sj* infection. Mice are sacrificed at week 8 after* Sj* infection and their liver or sera are collected. (a) IL-13 mRNA level in mouse liver of normal or* Sj*-infected mice with or without CTM treatment is detected by RT-PCR and Q-PCR. Upper panel: Q-PCR; lower panel: RT-PCR. GAPDH is detected as an internal control. Data are presented as mean ± SD from eight mice per group. **P < 0.01. (b) IL-13 concentration in mouse sera of normal or* Sj*-infected mice with or without CTM treatment is detected through ELISA. Data are presented as mean ± SD from 8 mice per group. *P < 0.05. (c) Mouse livers at indicated time points are fixed in paraformaldehyde, embedded in paraffin, sliced, and IHC stained for IL-13. Representative slices of IHC staining for IL-13 are shown at original 40x magnification.

CTM treatment can selectively inhibit the extent of tTG and liver fibrosis. Considering that IL-13 and tTG are involved in liver fibrosis after* Sj* infection, we tested the level change of IL-13 after CTM treatment. CTM showed a marked effect on IL-13 mRNA expression. While IL-13 was highly upregulated in* Sj*-infected mice, administration of CTM significantly decreased the mRNA expression of IL-13 in* Sj*-infected mouse livers (Figure 5(a)). Meanwhile, IL-13 level in peripheral venous blood was also decreased by 21.6% (Figure 5(b)). The alleviative effect was confirmed by IHC staining (Figure 5(c)). These results showed that CTM can downregulate IL-13 in case of* Sj* infection, at least partially, which provided a possibility that IL-13 expression can be upregulated by tTG.

## 4. Discussion

Liver fibrosis caused by* Sj* is one of the most serious pathological changes that may induce loss of liver function and liver cancer [26]. Owing to the early, aggregate, and massive egg-laying characteristics of* Sj*, liver fibrosis symptoms caused by* Sj* are the most grievous among those of the prevalent schistosomiasis. Hence, finding effective measures to prevent or even reverse liver fibrosis is critical in the battle against* Sj*. However, safe drug intervention will be impossible before we truly understand the immunopathogenesis and mechanism of liver fibrosis of this disease.

Our data showed the association of IL-13 and tTG with liver fibrosis. We elucidated the possible regulation of IL-13 expression by tTG as well. IL-13 is thought to be the key mediator of liver fibrosis in* Sm,* as antagonizing IL-13 or IL-13^−/−^ mouse liver fibrosis was abrogated [8, 9]. Although the correlation between IL-13 and liver fibrosis in* Sm* has been found, more research is required for further assessment of the mechanisms by which IL-13 influences liver fibrogenesis. Nevertheless, our findings serve as a basis for studying the function of IL-13 in infiltrating lymphocytes in case of* Sj* infection.

The function of tTG in liver fibrosis remains controversial. While most studies report the positive involvement of tTG in liver fibrosis, contradicting reports still exist. Most studies on the fibrogenesis function of tTG were performed on a carbon tetrachloride-induced liver fibrosis model [16, 17] and may thus fail to represent the fibrogenesis mechanism in many other pathological circumstances. In this study, we provided several lines of evidence suggesting that tTG accelerated* Sj*-induced liver fibrosis. First, tTG level was consistent with the extent of liver granuloma and fibrosis after* Sj* infection. tTG was mainly overexpressed in hepatic cells at week 5 and in the HSC at week 6 and mainly in the infiltrating cells around* Sj* eggs at weeks 8 and 12 (Figure 3). Second, the blocking of tTG activity by CTM treatment from days 3 to 10 after* Sj* infection alleviated liver fibrosis (Figure 4). The dose and the duration of CTM treatment were referred to in the literature [27, 28]. We tried to treat the infected mice at week 5 or 6 with CTM once a day for 7 d or 14 d, but the extent of fibrosis and also the level of tissue transglutaminase exhibited no change (data not shown). The underlying role and mechanisms of tTG involvement in the pathogenesis of* Sj* infection are unclear. These data suggest that HSC activation, as well as some inflammatory factors or secretory factors of* Sj* origin, may be the downstream targets of tTG. However, once the downstream pathways or cascades were initiated at the early stage of* Sj* infection, tTG inhibition could not reverse the pathologic changes. Further studies are needed to determine the detailed pathogenesis of tTG contribution to liver fibrosis after* Sj* infection.

The association of tTG with cytokines, such as IL-6, IL-17 [29], IL-33 [21], TGF-*β*1 [30], tumor necrosis factor alpha, and interferon gamma, had been reported [31]. tTG is an important inflammatory and fibrogenetic factor involved in Th17 [29] and Th2 responses [20]. We found that tTG partially regulates IL-13 in immunological response, but the underlying mechanisms of tTG regulation of IL-13 require further study.

Therefore, we propose a model to illustrate the possible mechanisms linking tTG, IL-13, and liver fibrosis. When exposed to repetitive damage (viral, parasitic, toxic, and metabolic), the liver reacts with a chronic wound healing response that usually results in fibrosis. Liver fibrogenesis is mainly driven by activated HSCs and an excess accumulation of extracellular matrix. The high extent of tTG expression and activity consequently activates NF-*κ*B signaling. The activated NF-*κ*B signaling pathway upregulates the production of some inflammatory cytokines, including IL-6, IL-17 [29], and IL-13. Meanwhile, by activating Th2 response [21], tTG may enhance the production of IL-13 by activating Th2. tTG inhibition through CTM reduces IL-13 and then suppresses liver fibrosis.

These findings suggest that both tTG and IL-13 can be drug targets for schistosomiasis liver fibrosis and that selective tTG inhibitory drug and downstream inhibition reagents may abrogate the liver fibrogenesis possibly by regulating IL-13. Further studies on the exact mechanisms of the tTG regulation of IL-13 during* Sj* infection are needed. However, we have to consider that the IL-13 level in peripheral venous blood was only decreased by 21.57%. Some compensatory mechanisms can possibly increase the level of IL-13 in the blood but not in the liver or some other factors aside from tTG may regulate IL-13 expression correlating with liver fibrosis after* Sj* infection.

## 5. Conclusion

Our data established a novel link among IL-13, tTG, and liver granuloma and fibrosis. The important function of IL-13 and tTG in* Sj* liver fibrosis makes them potential drug targets in preventing liver fibrogenesis.

## Supplementary Material

Total TGase activity in Liver protein lysate was measured by a modified nonradioactive microtiter plate assay. The results showed that TGase activity was markedly increased in the liver post Sj infection compared with uninfected mice (Figure S1). CTM-treated Sj-infected mice liver showed significantly lower TGase activity compared with those of untreated mice (Figure S2).
