# Supplementary material for: Involvement of IL-13 and Tissue Transglutaminase in Liver Granuloma and Fibrosis after Schistosoma japonicum Infection
Source: Mediators Inflamm. 2014 Jul 3;2014:753483. doi: 10.1155/2014/753483 (PMC4106180; doi:10.1155/2014/753483)
Supplement: Supplementary file 1 — Total TGase activity in Liver protein lysate was measured by a modified nonradioactive microtiter plate assay. The results showed that TGase activity was markedly increased in the liver post Sj infection compared with uninfected mice (Figure S1). CTM-treated Sj-infected mice liver showed significantly lower TGase activity compared with those of untreated mice (Figure S2). [file 753483.f1.docx]

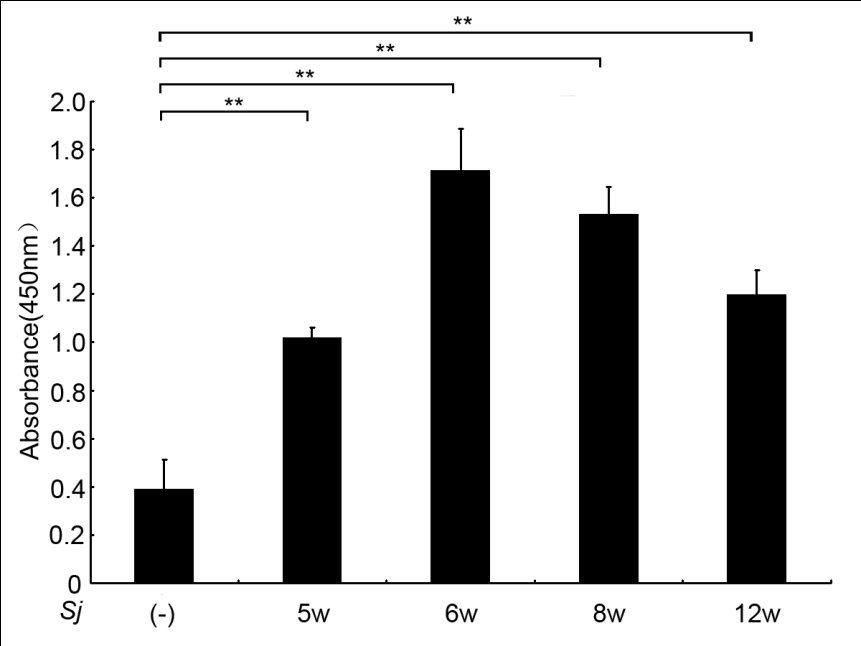


**Figure S1. Total transglutaminase (TGase) activity of mouse liver increased post *Sj* infection.** BALB/c mice were infected with 20±3 infective cercariae of *Sj* for 5, 6, 8, and 12 weeks, and non-infected mice served as negative control. Liver protein lysate is collected for TGase activity measurement. The amount of incorporated 5' (biotinamido) pentylamine was quantified by measuring the absorbance at 450 nm in a plate reader. Mean ± SD of 3 separate experiments. **P<0.01.


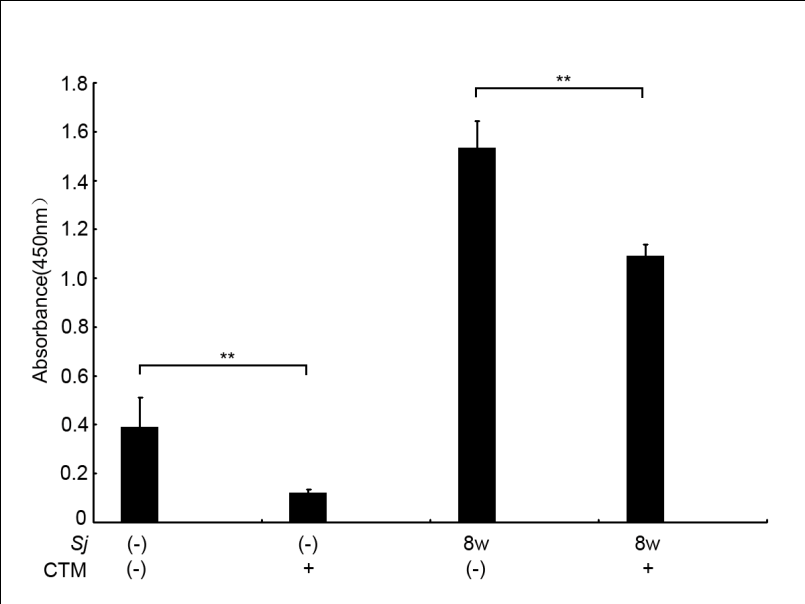


**Figure S2. CTM inhibited TGase activity of mouse liver.** TGase activity in BALB/c mice is blocked by CTM intraperitoneal injection from day 3 to day 10 post-*Sj* infection. Mice are sacrificed at week 8 post-infection. Liver protein lysate is collected for TGase activity measurement. The amount of incorporated 5' (biotinamido) pentylamine was quantified by measuring the absorbance at 450 nm in a plate reader. Mean ± SD of 3 separate experiments. **P<0.01.
